# Editorial: Immunomodulatory Functions of Nutritional Ingredients in Health and Disease

**DOI:** 10.3389/fimmu.2019.00050

**Published:** 2019-01-24

**Authors:** Jia Sun, Paul de Vos

**Affiliations:** ^1^State Key Laboratory of Food Science and Technology, Jiangnan University, Wuxi, China; ^2^School of Food Science and Technology, Jiangnan University, Wuxi, China; ^3^Division of Medical Biology, Department of Pathology and Medical Biology, University of Groningen, University Medical Center Groningen, Groningen, Netherlands

**Keywords:** nutritional intervention, gut microbiota, probiotics, dietary fibers, immune-related diseases

Nutritional immunology is a rapidly developing field. An expanding body of evidence demonstrate the impact of foods and nutritional components on gut and systemic immunity of consumers. During recent years, the implications of nutrition and nutritional intervention on prevention of disease have become accepted and has become an important tool in management of several diseases.

Nutritional immunology might become even more important in prevention of disease when the interplay between nutritional processes and immune system is better understood. Particularly, specific cellular and molecular immune responses provoked by nutrition and the role of the gut barrier and microbiota in the interplay needs more study.

This content of this Research Topic was designed to provide a timely collection on mechanistic, translational, and clinical research on the interplay between foods, nutritional components, and immunity in physiological and pathophysiological conditions.

We have a series of original or review articles featuring the role of gut microbiota impacted by various factors, specific probiotics in shaping immunity and implications in immune inflammatory diseases.

Fransen et al. focused on aged gut microbiota and health. Advanced age is associated with chronic low-grade inflammation, referred to as inflammaging. The elderly are also known to harbor an altered gut microbiota composition. It was unknown whether this altered gut microbiota was cause or consequence of inflammaging. To this end, Fransen et al. performed microbiota transfer from old mice to germ free young mice and demonstrated that the gut microbiota from old mice contributes to inflammaging after transfer to young GF mice. This knowledge might lead to targeted strategies to juvenile the microbiota in elderly to reduce inflammaging.

Besides aging, gender affects the immune system and gut microbiota composition. Also here cause or consequence was unknown. In this context, the same group demonstrate that gender differences in immunity are already present in GF mice, independent of gut microbiota and that microbiota-independent gender differences in the immune system select a gender-specific gut microbiota composition, which in turn further contributes to gender differences in the immune system (Fransen et al.). This research suggests that modulation of immunity by nutrition might need a gender specific approach.

Accumulating evidence supports an important role of diet and gut microbiota in immune-mediated diseases. Multiple sclerosis (MS) is an autoimmune neurological disease characterized by chronic inflammation of the central nervous system (CNS), leading to demyelination, axonal damage, and symptoms such as fatigue and disability. The infiltration of peripherally activated immune cells into the CNS has a key pathogenic role. Preclinical as well as clinical studies suggest a role for gut microbiota and dietary components in MS. The review from van Den Hoogan et al. focused on recent studies on gut microbiota and dietary interventions in MS and prospective animal models in which efficacy of dietary intervention can be tested.

In this Research Topic, we have also several studies focusing on different specific probiotic strains to modulate immune-related conditions. An imbalance in gut microbiota composition can lead to impaired intestinal homeostasis, chronic gut inflammation and a predisposition to developing, for example, colorectal cancer (CRC). The use of probiotic bacteria has emerged as an additive strategy to treat and prevent cancer. Moreover, consumption of beneficial bacteria may favorably modulate the composition of gut microbiota, which has been described in several studies to play an important role in preventing CRC carcinogenesis. In this regard, Jacouton et al. assessed the protective effect of oral treatment with *Lactobacillus casei* BL23, a probiotic strain well-known for its anti-inflammatory and anticancer properties in CRC. Their results demonstrate high potential of *L. casei* BL23 for the development of new, probiotic-based strategies to fight CRC.

Oral bacteria interact with intestinal mucosa and impact immunity. However, mechanistic insights are limited. de Vos et al. conducted a randomized placebo-controlled cross-over trial, to evaluate *Lactobacillus plantarum* supplementation (strain TIFN101, CIP104448, or WCFS1) or placebo in healthy human subjects for 7 days. Their data show that specific bacterial strains can prevent immune stress induced by commonly consumed painkillers such as non-steroidal anti-inflammatory drug (NSAID) and can have enhancing beneficial effects on immunity of consumers by stimulating antigen presentation and memory responses. This study demonstrates that probiotic species can serve as a mean to prevent side-effects of medication.

Indigenous *Clostridium* species have been recently demonstrated to induce colonic regulatory T cells (Tregs), and gut lymphocytes are able to migrate to pancreatic islets in an inflammatory environment. Jia et al. investigated whether supplementation with the well-characterized probiotics *Clostridium butyricum* CGMCC0313.1 (CB0313.1) may induce pancreatic Tregs and consequently dampen the diabetes incidence in non-obese diabetic (NOD) mice. This study provide the basis for future clinical investigations in preventing type 1 diabetes (T1D) by oral CB0313.1 administration.

In another animal model for T1D, in biobreeding diabetes-prone rats, it has been demonstrated that *Lactobacillus johnsonii* N6.2 mitigated the onset of diabetes, in part, through changes in kynurenine:tryptophan (K:T) ratios. As a step toward human application, Marcial et al. performed a pilot double-blind, randomized clinical trial to determine the safety, tolerance, and general immunological response of *L. johnsonii* N6.2 in healthy subjects. Forty-two healthy individuals with no known risk factors for T1D were involved to evaluate subject responses to the consumption of *L. johnsonii* N6.2. The data provide support for the safety and feasibility of using *L. johnsonii* N6.2 in prevention trials in subjects at risk for T1D.

Acute pancreatitis (AP) is a common abdominal inflammatory disorder and one of the leading causes of hospital admission for gastrointestinal disorders. No specific pharmacological or nutritional therapy is available but highly needed. In this Research Topic, Pan et al. provided a comprehensive review on recent advances on nutritional treatment of acute pancreatitis, focusing on enteral vs. parenteral nutrition strategies, and nutritional supplements such as probiotics, glutamine, omega-3 fatty acids, and vitamins in clinical AP. The review gives several leads to successful nutrition and nutritional supplements for clinical management of AP.

In addition, the same group have focused on dietary fibers in preventing or treating AP. Inulin-type fructans (ITFs) are capable of modifying gut immune and barrier homeostasis in a chemistry-dependent manner and hence potentially applicable for managing AP, but their efficacy in AP has not been demonstrated yet. He et al. examined and compared modulatory effects of ITFs with different degrees of fermentability on pancreatic-gut immunity and barrier function during experimentally induced AP in mice. The results demonstrate a clear chain length-dependent effect of inulin to alleviate AP.

Obesity and metabolic syndrome are currently recognized as worldwide epidemics that pose a profound socioeconomic impact and represent a concern to public health. Cells of the immune system contribute to both the maintenance of “lean homeostasis” and metabolic dysregulation observed in obese individuals. Mounting evidence suggest that food additives may also be important contributors to metabolic derangement. The latest review from Paula Neto et al. summarizes latest literature evidence that food additives have relevant effects on cells of the immune system that could contribute to immune-mediated metabolic dysregulation. The reviewed data suggest that some food additives should be avoided considering the adverse effects of these additives to predispose individuals to develop obesity and metabolic syndrome.

In another aspect, sexual dimorphism in immune response is widely recognized, but few human studies have observed this distinction. Food with endo-immunomodulatory potential may reveal novel sex-biased *in vivo* interactions. To this end, Jumat et al. compared immunomodulatory effects of *Carica papaya* compared between healthy male and female individuals. Their data show clear dissimilar immune profiles are elicited in the sexes after papaya consumption and may have sex hormone influence.

Lastly, we have two articles focusing on nutrient effects on health and diseases. Excessive sodium intake is often associated with high risk for cardiovascular disease. More recently, high-salt diets (HSDs) have been demonstrated to activate Th17 cells and increase severity of autoimmune diseases. Aguiar et al. evaluated the effects of a diet supplemented with NaCl in the colonic mucosa at steady state and during inflammation. They found that consumption of HSD *per se* triggered a histologically detectable inflammation in the colon and also exacerbated chemically induced models of colitis in mice by a mechanism dependent on IL-17 production most likely by both ILC3 and Th17 cells.

L-arginine deficiency is shown to be associated with a growing number of diseases in humans, including trauma, certain cancers, and infection. L-arginine supplementation is essential during pregnancy to support fetal development. In conditions of l-arginine depletion, T cell proliferation is impaired. Previously, it has been shown that neonatal blood has lower l-arginine levels than adult blood, which is associated with poor neonatal lymphocyte proliferation, and that l-arginine enhances neonatal lymphocyte proliferation through an interleukin (IL)-2-independent pathway. Yu et al. have further investigated how exogenous l-arginine enhances neonatal Treg function in relation to IL-10 production under epigenetic regulation. Their results suggest that l-arginine modulates neonatal Tregs through the regulation of IL-10 promoter DNA methylation. L-arginine supplementation may correct the Treg function in newborns with l-arginine deficiency.

The scientific contributions collectively show the important role of dietary components in immune homeostasis and the potential of specific food ingredients to prevent disease or manage disease symptoms. Insights in how specific food components might impact gut microbiota, barrier function or immunity-receptors might lead to targeted and rationally designed strategies to avoid immune related diseases. At the same time, the Research Topic shows that some ingredients may have adverse effects and should be avoided in sensitive subjects and that targeted groups such as different genders and age-classes have to be distinguished for optimal efficacy of nutritional interventions.

## Author Contributions

All authors listed have made a substantial, direct and intellectual contribution to the work, and approved it for publication.

### Conflict of interest Statement

The authors declare that the research was conducted in the absence of any commercial or financial relationships that could be construed as a potential conflict of interest.

